# Medical equipment effectiveness evaluation model based on cone-constrained DEA and attention-based bi-LSTM

**DOI:** 10.1038/s41598-024-59852-4

**Published:** 2024-04-23

**Authors:** Luying Huang, Wenqian Lv, Qingming Huang, Haikang Zhang, Siyuan Jin, Tong Chen, Bing Shen

**Affiliations:** 1 Shanghai General Hospital, Shanghai Jiao Tong University School of Medicine, Shanghai, 200080 China; 2 School of Health Science and Engineering, University of Shanghai for Science and Technology, Shanghai, 200093 China; 3 School of Medical Imaging, Shanghai University of Medicine and Health Sciences, Shanghai, 201318 China; 4 Shanghai Tenth People’s Hospital of Tongji University, Shanghai, 200072 China

**Keywords:** Data envelopment analysis, Decision-making methods, Medical equipment, Effectiveness evaluation, Attentional Bi-LSTM, Applied mathematics, Biomedical engineering

## Abstract

This study constructs a composite indicator system covering the core dimensions of medical equipment input and output. Based on this system, an innovative cone-constrained data envelopment analysis (DEA) model is designed. The model integrates the advantages of the analytic hierarchy process (AHP) with an improved criterion importance through intercriteria correlation (CRITIC) method to determine subjective and objective weights and employs game theory to obtain the final combined weights, which are further incorporated as constraints to form the cone-constrained DEA model. Finally, a bidirectional long short-term memory (Bi-LSTM) model with an attention mechanism is introduced for integration, aiming to provide a novel and practical model for evaluating the effectiveness of medical equipment. The proposed model has essential reference value for optimizing medical equipment management decision-making and investment strategies.

## Introduction

With the advancement of medical technology, efficient diagnosis and treatment rely heavily on sophisticated medical equipment, which is also a significant investment consideration for health care institutions. Efficient diagnosis and treatment in health care rely heavily on advanced medical equipment^1^. Therefore, it is important for health care organisations to invest in such equipment. Regular evaluations are essential to keep up with rapid technological advances and to ensure that health care institutions are equipped with efficient and up-to-date equipment. This not only justifies investment in medical equipment but also ensures the sustainability of medical practices. In short, evaluating the effectiveness of medical equipment is essential for improving the quality of care, ensuring cost effectiveness, optimizing the allocation of health care resources, and making informed strategic decisions^2^.

Multicriteria decision analysis (MCDA) techniques^3^, including data envelopment analysis (DEA)^4^, the analytic hierarchy process (AHP)^5^, and the enhanced CRITIC method^6^, provide new perspectives for medical equipment efficiency evaluation by constructing quantifiable attribute indicator systems^7^ that can provide a basis for ranking the advantages and disadvantages of different aspects of equipment.

Since its introduction by Charnes et al. in 1978^8^, data envelopment analysis (DEA) has been extensively applied across various domains for efficiency evaluation because of its ability to handle multiple inputs and outputs. DEA extends the concept of single-input, single-output technical efficiency to multiple-input, multiple-output relative efficiency evaluations in the CCR model. By analysing the data from the input‒output indicators, the CCR model can identify efficient decision-making units (DMUs), reveal the reasons for and extent of other noneffective decision-making units, and provide efficient information for improvement^9^.

However, as the study progressed, traditional DEA models may have limitations in addressing outlier data points, environmental influences, and nonradial efficiency differences among DMUs. Extended DEA models, such as cone-ratio DEA^10^, statistical inference^11^, and multistage DEA^12^, which incorporate additional constraints suitable for different objectives, have been developed to enhance the robustness of DEA. Du used an additive superefficiency DEA model to measure the efficiency of 119 general acute care hospitals in Pennsylvania, USA, using a set of seven inputs and outputs^13^. Dehghan P used the superefficiency model to find the most efficient Siemens MRI machine and ranked the available models using DEA^14^. Yaya S established an ingenious and efficient model that combines weighted data envelopment analysis (DEA) and game theory to improve the quality and management of health care^15^. Hajiagha et al. proposed a three-stage approach to data envelopment analysis (DEA) modelling for hospital efficiency, which aims to overcome the constraint on the number of inputs/outputs relative to the number of DMUs^16^. Xie combined the indicator system of DEA based on the entropy weight idea, obtained the corresponding indicator weights, and finally assembled the results^17^. Amindoust Atefeh combined fuzzy set theory with DEA from a sustainable perspective, taking into account the preferences of decision-makers and dealing with ambiguity and uncertainty in the supplier selection process^18^. Mohamed combined data envelopment analysis (DEA) and analysis of hierarchy (AHP) to measure the weights of efficiency factors based on pairwise comparisons of neutral language scales and 11 factors; this approach was then used to measure the efficiency of emergency departments in 20 hospitals^19^.

On the other hand, recurrent neural networks (RNNs)^20^, incredibly long short-term memory (LSTM) models, have gained popularity because of their superior ability to process time series data. Unlike feedforward neural networks, RNNs can handle sequential dependencies by allowing information to persist and circulate across network recursions, thus capturing dynamic patterns in temporal data. The gating mechanisms in LSTMs further enable selective memorization and forgetting of time series long-range dependencies. Moreover, bidirectional LSTM with an attention mechanism^21^ can better identify critical points in time series by dynamically reweighting time steps, thus improving the prediction accuracy and robustness.

Although current methods have made some progress in evaluating objects, existing methods still have a few shortcomings. Firstly, traditional evaluation methods tend to focus on a single criterion or method, lacking a comprehensive evaluation of the object under consideration. Second, some of the existing DEA models have limitations in dealing with complex health care environments and diverse efficiency factors. For example, traditional DEA models cannot reflect the objective differences in importance between indicators and the preferences of decision-makers; DEA models with AHP constraint cones are not uniform in their scalar evaluation; and superefficiency DEA models remove specific decision-making units from their calculations, making them less comprehensive.

Therefore, this study proposes an innovative evaluation model that integrates the analytic hierarchy process (AHP), improved CRITIC, game theory^22^, cone-constrained DEA, and attention-based Bi-LSTM to enable a more comprehensive and accurate evaluation of medical equipment effectiveness. By synergistically applying these advanced techniques, the model aims to provide a thorough assessment to facilitate evidence-based decision-making regarding medical equipment management and investment.

## Establishment of a system for evaluating the effectiveness of medical equipment

This study develops a medical equipment effectiveness evaluation system to help health care facilities and related departments manage and update medical equipment more effectively^23^. The goals are to ensure high performance, reduce operating costs, and improve health care service quality. The system construction incorporates the target hospital's specific requirements and the equipment's technical characteristics. Moreover, evaluation model development follows systematic, operable, independent, and quantifiable principles^24^.

### Input indicators

(1) Equipment utilization rate $\left({X}_{1}\right)$: The equipment utilization rate reflects the equipment load in operation. Higher operating saturation may lead to overworked equipment, affecting its stability and reliability.

1 $$X_{1} = \frac{{\left( {\tau + 2} \right) \times \sum Z_{i} }}{{\sigma_{b} }}$$

where $\tau$ is the average examination time per patient, $Z$ is the total number of inspections, and ${\sigma }_{b}$ is the energetic running time of the equipment. 2 is the time set aside for preparation.

(2) Average daily working hours $\left({X}_{2}\right)$: The average daily working hours is the number of hours the equipment works continuously per day.

2 $$X_{2} = \sum \left( {t_{e} - t_{s} } \right)$$

where ${t}_{e}$ and ${t}_{s}$ represent the end time of the patient's examination and the start time of the examination, respectively.

(3) Equipment operating costs $\left({X}_{3}\right)$: The equipment operating costs include energy, maintenance, repair, and personnel costs and represent the cost of inputs to keep the equipment in operation.

(4) Average cost per inspection $\left({X}_{4}\right)$: The average cost per inspection reflects the cost per inspection when the patient uses the equipment.

3 $$X_{4} = \frac{{C_{t} + P}}{{\sum Z_{i} }}$$

where ${C}_{t}$ is the equipment operating cost and $P$ is the equipment depreciation cost.

### Output indicators

(1) Inspection revenue $\left({X}_{5}\right)$: Inspection revenue is the total revenue generated from equipment inspections and represents the total output of the equipment. Higher inspection revenue indicates greater equipment efficiency.

(2) Average Patient Appointment Waiting Time $\left({X}_{6}\right)$: The average patient appointment waiting time is the time between a patient's appointment and the actual examination, which can reflect the timeliness of equipment use.

4 $$X_{6} = \frac{{\sum \left( {\tau_{bi} - \tau_{ci} } \right)}}{{\sum Z_{i}{\prime} }}$$

where ${\tau }_{bi}$ refers to the start time of the patient's examination, ${\tau }_{ci}$ refers to the patient's appointment time, and ${Z}_{i}^{\mathrm{^{\prime}}}$ refers to the number of patients.

(3) Cost‒benefit ratio $\left({X}_{7}\right)$: The cost‒benefit ratio refers to the relationship between the revenue generated by the equipment and the operating cost, which helps to explain the input‒output capability.

5 $$X_{7} = \frac{{R_{t} }}{{C_{t} { }}}$$

where ${R}_{t}$ refers to the inspection revenue and ${C}_{t}$ refers to the equipment operating cost.

(4) Waiting time for examination reports $\left({X}_{8}\right)$: The waiting time for examination reports is the amount of time a patient waits for an examination report. Shorter times reflect greater societal benefits from the use of the equipment.

6 $$X_{8} = \frac{{\sum \left( {\gamma_{bi} - \gamma_{ci} } \right)}}{{\sum Z_{i}{\prime} }}$$

where ${\gamma }_{bi}$ refers to the time at which the patient receives the report, and ${\gamma }_{ci}$ refers to the time at which the examination of the patient is completed.

## Materials and methods

### General DEA model

The DEA model is derived from the idea of relative efficiency, which is to evaluate the relative efficiency of a multi-input and multioutput system based on linear programming^25^. The degree of efficiency of each decision unit is compared to determine the degree of efficiency of each decision unit.

7 $$h_{j} = \frac{{u^{T} y_{j} }}{{v^{T} x_{j} }} = \frac{{\mathop \sum \nolimits_{r = 1}^{s} u_{r} y_{rj} }}{{\mathop \sum \nolimits_{i = 1}^{m} v_{i} x_{ij} }}$$

where $j$ is the evaluation object, known as the decision unit; there are *n* decision-making units, so $j=\mathrm{1,2},\dots ,n$. $i$ is a total of $m$ input indicators, $i=\mathrm{1,2},\dots ,m$. $r$ is a total of $s$ output indicators, $r=\mathrm{1,2},\dots ,s$. ${x}_{ij}$ refers to the number of $i$ input indicators of the $j$ decision unit, ${x}_{ij}>0$. ${y}_{rj}$ refers to the value of the $r$ output indicator of the $j$ decision unit. ${v}_{i}$ refers to the weight of the $i$ input indicator. ${u}_{r}$ refers to the weight of the $r$ output indicator.

8 $$v = \left( {v_{1} ,v_{2} , \ldots ,v_{m} } \right)^{T} ,u = \left( {u_{1} ,u_{2} , \ldots ,u_{s} } \right)^{T}$$

The optimal model is constructed as follows:

9 $$\left\{ {\begin{array}{*{20}l} {maxh_{j0} = \frac{{u^{T} y_{j0} }}{{v^{T} x_{j0} }}} \hfill \\ {h_{j} = \frac{{u^{T} y_{j} }}{{v^{T} x_{j} }} \le 1} \hfill \\ {v = \left( {v_{1} ,v_{2} , \ldots ,v_{m} } \right)^{T} \ge 0} \hfill \\ {u = \left( {u_{1} ,u_{2} , \ldots ,u_{s} } \right)^{T} \ge 0} \hfill \\ \end{array} } \right.$$

Let $\text{t} = \frac{1}{{v}^{T}{x}_{0}}, w=tv, {\text{and}} \mu =tu$, which can be transformed into the following:

10 $$\left\{ {\begin{array}{*{20}l} {maxh_{j0} = \mu^{T} y_{j0} } \hfill \\ {w^{T} x_{j} - \mu^{T} y_{j} \ge 0} \hfill \\ {w^{T} x_{j0} = 1} \hfill \\ {w\left( {w_{1} ,w_{2} , \ldots ,w_{m} } \right)^{T} \ge 0} \hfill \\ {\mu \left( {\mu_{1} ,\mu_{2} , \ldots ,\mu_{s} } \right)^{T} \ge 0} \hfill \\ \end{array} } \right.$$

### Determination of combinationed weights

#### AHP

The analytic hierarchy process (AHP)^26^ is a systematic and hierarchical multicriteria decision-making technique. AHP models complex decision problems with hierarchical objectives, criteria, and alternatives^27^. It then quantitatively determines the relative importance of elements at each level through pairwise comparisons, leading to the calculation of criteria weights. Due to its ability to incorporate qualitative and quantitative factors in a simple hierarchical model, the AHP has been extensively applied in areas such as resource allocation, project appraisal, and strategy planning.

*Step 1*: Construct a judgement matrix. Two-by-two comparisons are performed based on the relative importance of the hierarchical indicators on a 1–9 scale:

11 $$V_{i} = \sqrt[n]{{\mathop \prod \limits_{i = 1}^{n} A_{ij} }}$$

where $i$ and $j$ refer to the ordinal numbers of the rows and columns of the matrix, respectively, and $n$ is the number of indicators in the judgement matrix.

*Step 2*: Weight calculation. The weight value of each indicator can be obtained by calculating the eigenvectors of the judgement matrix.

12 $$W_{i}^{A} = \frac{{V_{i} }}{{\mathop \sum \nolimits_{i = 1}^{n} V_{i} }}$$

*Step 3*: Consistency test. Consistency tests are required to ensure the consistency of judgements.

13 $${\text{CI}} = \frac{{\lambda_{max} - n}}{n - 1}$$

where the most prominent characteristic root is ${\lambda }_{max}=\sum_{i-1}^{n}\frac{{\left({\text{BW}}\right)}_{i}}{n{W}_{i}^{A}}$, and ${\left({\text{BW}}\right)}_{i}$ is the product of the judgement matrix and indicator weights.

#### Improvement of the CRITIC Method

The criteria importance through intercriteria correlation (CRITC) method^28^ proposed by Diakoulaki is an objective weighting technique based on information entropy theory^29^. The attribute weights are determined by comparing the information content between criteria. Specifically, intercriteria correlation is first calculated to measure information similarity. Attributes with lower correlations to others contain more unique information and thus have higher weights. By considering information entropy and interdependence, CRITIC provides a more objective approach to criteria weighting than do subjective rating methods.

*Step 1*: Using the initial sample data, the indicator values are standardized according to the deviation standardization method to obtain a standardized decision matrix $Z={\left({z}_{ij}\right)}_{m\times n}$ that is used to find the combined information of each indicator based on the distance ${f}_{j}{\prime}$:

14 $$f_{j}{\prime} = h_{j} \mathop \sum \limits_{i = 1}^{m} \left( {1 - r_{ij} } \right)$$

where ${r}_{ij}$ is the difference between the correlation coefficient of the $i$ indicator and the $j$ indicator. ${h}_{j}$ is the standard deviation of the $j$ indicator.

*Step 2*: The weight of each indicator ${W}_{j}$ is calculated:

15 $$W_{j} = \frac{{f_{j}{\prime} }}{{\mathop \sum \nolimits_{i = 1}^{n} f_{j}{\prime} }}$$

Improving upon the CRITIC method, since ${r}_{ij}$ has positive and negative correlation coefficients with the same absolute value, the correlation degree between the indicators should be the same, so $\left(1-\left|{r}_{ij}\right|\right)$ is used instead of $\left(1-{r}_{ij}\right)$, which is used in the original method. Moreover, in terms of the intensity of comparison and the degree of conflict, the CRITIC method is improved to consider the degree of dispersion between the indicator data. Therefore, the attributes of the indicator data can be fully considered, and the formula of the improved CRITIC method is calculated as follows:

16 $$W_{j}^{c} = \frac{{\left( {\sigma_{j} + h_{j} } \right)\mathop \sum \nolimits_{i = 1}^{m} \left( {1 - \left| {r_{ij} } \right|} \right)}}{{\mathop \sum \nolimits_{j = 1}^{n} \left[ {\left( {\sigma_{j} + h_{j} } \right)\mathop \sum \nolimits_{i = 1}^{m} \left( {1 - \left| {r_{ij} } \right|} \right)} \right]}}$$

where ${\sigma }_{j}$ is the indicator determined by entropy method $j$.

#### Game theory weights

This study introduces game theory for weight aggregation to integrate the strengths of different weight determination techniques and enhance the objectivity, scientificity, and accuracy of combined weights^30^. Game theory investigates the decision-making of players in competitive and cooperative scenarios. It models each method as a player, weights the values as strategies, and finds the optimal strategy combination by solving the Nash equilibrium^31^. Different weighting approaches can be viewed as game players in multicriteria decision analysis. The equilibrium solution yields scientifically integrated weights for the decision problem by modelling their weight vectors as strategies.

*Step 1*: Set up a total of $L$ methods to determine the weights of $n$ indicators; the indicator weights are ${W}_{G}=\left({W}_{{G}_{1}},{ W}_{{G}_{2}}{,\dots ,W}_{{G}_{n}}\right)$; then, the combined weight $W$ is:

17 $$W = \mathop \sum \limits_{k = 1}^{L} \lambda_{G} W_{G} , G = 1,2, \ldots ,L$$

where ${\lambda }_{G}$ is the linear combination coefficient.

*Step 2*: Construct the optimal model to minimize the divergence $\Delta =\left(W-{W}_{G}\right)$ between $W$ and ${W}_{G}$:

18 $$min\mathop \sum \limits_{k = 1}^{L} \left| {\left| {\lambda_{G} W_{G}^{T} - W_{G} } \right|} \right|_{2} , G = 1,2, \ldots ,L$$

*Step 3*: Solve the game equilibrium. This study uses two weighting methods, taking the value L = 2. According to the principle of differentiation, the system of linear equations for the optimal first-order derivative condition is obtained as follows:

19 $$\left[ {\begin{array}{*{20}c} {W_{1} W_{1}^{T} } & {W_{1} W_{2}^{T} } \\ {W_{2} W_{1}^{T} } & {W_{2} W_{2}^{T} } \\ \end{array} } \right]\left[ {\begin{array}{*{20}c} {\lambda_{1} } \\ {\lambda_{2} } \\ \end{array} } \right] = \left[ {\begin{array}{*{20}c} {W_{1} W_{1}^{T} } \\ {W_{1} W_{2}^{T} } \\ \end{array} } \right]$$

*Step 4*: Find the combination coefficients ${\lambda }_{1}$ and ${\lambda }_{2}$, and obtain the game combination weight ${W}^{G}$ after normalization:

20 $$W^{G} = \lambda_{1} W_{1}^{T} + \lambda_{2} W_{2}^{T}$$

### Cone-constrained DEA

In conventional data envelopment analysis (DEA), the absence of weight constraints on input and output variables compromises the model's ability to reflect the decision-maker's preferences^32,33^. Chames et al. proposed the cone-constrained DEA model to address this limitation, which reflects the decision weights by adjusting the cone ratio^34,35^.

This advance incorporates decision weights by adjusting cone ratios, providing a more nuanced evaluation model. In contrast, the general DEA model is advantageous for assessing the relative efficiency among similar decision-making units (DMUs), which has notable shortcomings such as subjective weight selection and limited adaptability to anomalous datasets or complex decision environments. To strengthen the robustness and reliability of DEA-based evaluations, the cone-constrained DEA model has been further developed by incorporating diverse constraints to facilitate the treatment of nonstandard data.

*Step 1*: Construct the judgement matrix ${W}_{A}$, ${W}_{C}$. Suppose there are $N$ decision-making units, the input and output indicators are $m$ and $s$, respectively, and the combination weights are substituted into the matrix:

21 $$W_{A} = \left[ {\begin{array}{*{20}c} {W_{12}^{A} } & \cdots & {W_{m1}^{A} } \\ \vdots & \ddots & \vdots \\ {W_{1m}^{A} } & \cdots & {W_{mm}^{A} } \\ \end{array} } \right]$$

22 $$W_{C} = \left[ {\begin{array}{*{20}c} {W_{12}^{C} } & \cdots & {W_{s1}^{C} } \\ \vdots & \ddots & \vdots \\ {W_{1s}^{C} } & \cdots & {W_{ss}^{C} } \\ \end{array} } \right]$$

*Step 2*: Calculate the weight vector. Let ${\lambda }_{A}$ and ${\lambda }_{C}$ be the largest eigenvalues of the judgement matrices ${W}_{A}$ and ${W}_{C}$; then, the weight vectors $B$ and $C$ are as follows:

23 $$B = W_{A} - \lambda_{A} E_{m} , C = W_{C} - \lambda_{C} E_{s}$$

where ${E}_{m}$ and ${E}_{s}$ are the unit metrics of the input metrics of order $m$ and the output metrics of order $s$, respectively.

*Step 3*: Construct the constraint cone.

24 $$\begin{gathered} V = \left\{ {Bw \ge 0,w = \left( {w_{1} ,w_{2} , \ldots ,w_{m} } \right)^{T} \ge 0} \right\} \hfill \\ U = \left\{ {C\mu \ge 0,\mu = \left( {\mu_{1} ,\mu_{2} , \ldots ,\mu_{s} } \right)^{T} \ge 0} \right\} \hfill \\ \end{gathered}$$

The constraint cones are incorporated as constraints in ${{\text{C}}}^{2}{\text{WH}}$ into the model:

25 $$\left\{ {\begin{array}{*{20}l} {max\mu^{T} y_{j0} = V_{p} } \hfill \\ {w^{T} x_{j} - \mu^{T} y_{j} \ge 0} \hfill \\ {w^{T} x_{j0} = 1} \hfill \\ {w,\mu \ge 0} \hfill \\ {\mu \in U,w \in V} \hfill \\ \end{array} } \right.$$

### Attention-based Bi-LSTM

**Figure 1:**
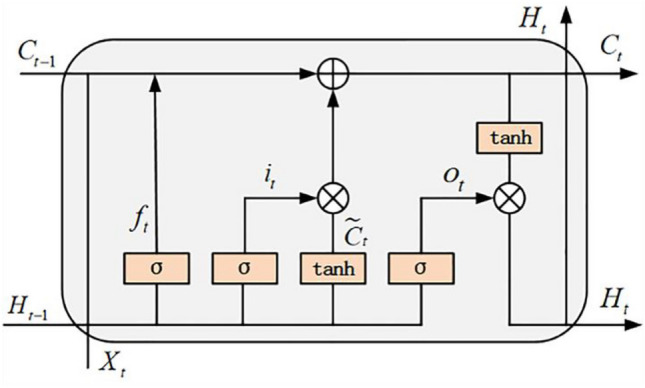
LSTM Model.

A long short-term memory (LSTM) neural network^36^ typically consists of an input layer, one or more hidden LSTM layers, and an output layer. An LSTM block mainly contains storage unit states, forget gates, and input and output gates, where the storage unit states are the critical elements of the whole LSTM block. Moreover, certain information can be added and removed with the help of these three gates. In Fig. 1, the input feature $x$ first passes through the gating structure of the forget gate and input gate to process the incoming new information by controlling the retention and forgetting of the information. At the same time, the hidden state $H$ participates in this process to make the LSTM unit memory capable^37^. The activation functions $\sigma$ and $tanh$ are sigmoid and hyperbolic tangent functions between 0 and 1, respectively, which enhance the nonlinearity of the artificial neural network.

The bidirectional long short-term memory (Bi-LSTM) network^38^ comprises two LSTM components— and backward LSTMs—to capture both past and future information of input sequences. The forward LSTM processes the sequence chronologically, encoding the past context in its hidden state, while the backward LSTM operates in reverse order to encode the future context. The final outputs concatenate representations from both directions, incorporating sequential dependencies to learn global patterns. Compared with unidirectional LSTMs, Bi-LSTMs can better model the contextual relationships and long-range dependencies of time series data^39^.

Given an input sequence $X=\left({x}_{1}\text{,}{x}_{2},\cdots ,{x}_{T}\right)$ where $T$ is the sequence length, Bi-LSTM processes the sequence independently through two directions. The forward LSTM, denoted as ${LSTM}^{f}$, encodes inputs chronologically from $t=1$ to $T$, and outputs hide the state ${H}_{t}^{f}$ at each step $t$. Meanwhile, the backward LSTM, denoted as ${LSTM}^{b}$, encodes inputs chronologically from $t=T$ to $t$, and outputs hide the state ${H}_{t}^{b}$:

26 $$\begin{gathered} H_{t}^{f} = LSTM^{f} \left( {x_{t} ,H_{t - 1}^{f} } \right) \hfill \\ H_{t}^{b} = LSTM^{b} \left( {x_{t} ,H_{t - 1}^{b} } \right) \hfill \\ \end{gathered}$$

The forward and backwards hidden states are combined to obtain information about the entire sequence at each time point:

27 $$H_{t} = H_{t}^{f} \oplus H_{t}^{b}$$

**Figure 2:**
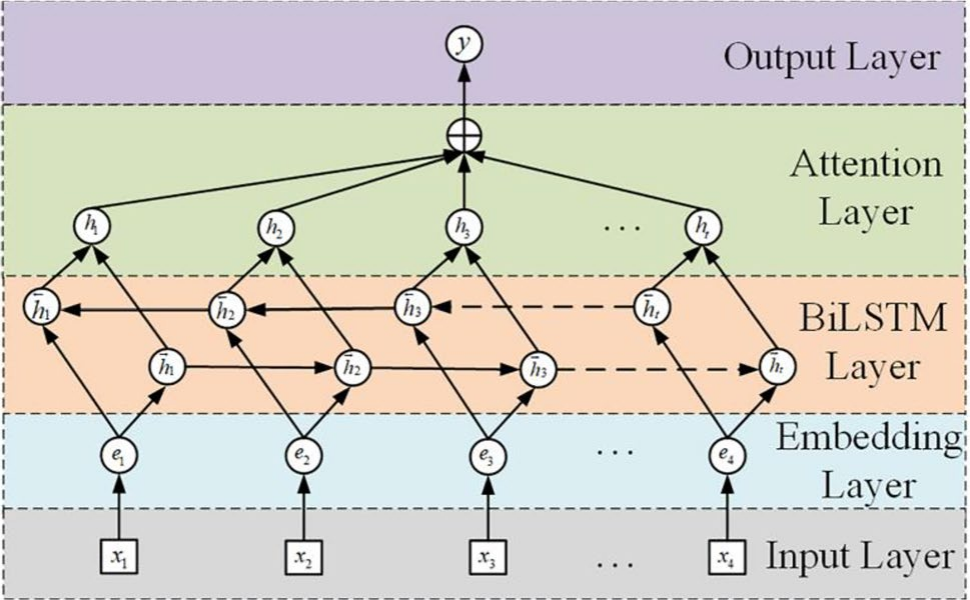
Attention-based Bi-LSTM Model.

Building on Bi-LSTM networks, integrating attention mechanisms^40^ can further enhance the identification and modelling of critical features in time series (Fig. 2), thereby improving the temporal prediction task performance. Attention is designed to assign different weights to inputs, selectively focusing on more informative elements. During implementation, the Bi-LSTM first encodes the input sequence^41^. The attention module automatically decodes the most discriminative states based on the prediction goal. The attention-enhanced Bi-LSTM demonstrates a superior ability to extract essential temporal signals.

For the time step $t$, the attention mechanism computes the weighting of the model input sequence ${\alpha }_{t, i}$:

28 $$\alpha_{t,i} = \frac{{exp\left( {score\left( {s_{t} ,H_{i} } \right)} \right)}}{{\mathop \sum \nolimits_{j = 1}^{T} exp\left( {score\left( {s_{t} ,H_{j} } \right)} \right)}}$$

where ${s}_{t}$ is the hidden state at the current output time step, and the score is used to calculate the similarity between two hidden states.

The weighted context sector ${c}_{t}$ is a weighted representation of the whole sequence, where higher weights are given to the parts more relevant to the current time step. Combining it with the original hidden state produces an attention-enhanced ${H}_{i}{\prime}$:

29 $$c_{t} = \mathop \sum \limits_{i = 1}^{T} \alpha_{i} H_{i}$$

30 $$H_{t}{\prime} = c_{t} + H_{t}$$

After constructing the LSTM neural network model, its performance needs to be tested. The prediction correlation coefficient ${R}^{2}$, root mean square error (RMSE), relative error (RE), and mean absolute percentage error (MAPE) between the predicted and measured values were compared. The formulae are as follows:

31 $$R^{2} = 1 - \frac{{\mathop \sum \nolimits_{i = 1}^{n} \left( {M_{i} - P_{i} } \right)^{2} }}{{\mathop \sum \nolimits_{i = 1}^{n} \left( {M_{i} - \overline{M}} \right)^{2} }}$$

32 $$RMSE = \sqrt {\frac{1}{n}\mathop \sum \limits_{i = 1}^{n} (M_{i} - P_{i} )^{2} }$$

33 $$MAPE = \left( {\frac{{\mathop \sum \nolimits_{i = 1}^{n} \left| {M_{i} - P_{i} } \right|}}{{nM_{i} }}} \right)$$

## Case studies

### Case descriptions and data sources

In the current climate where health care services are increasingly limited, magnetic resonance imaging (MRI) is an indispensable diagnostic tool that plays a pivotal role in accurately diagnosing diseases and formulating treatment plans. However, the considerable initial investment and substantial ongoing operational and maintenance costs make MRI systems a critical focal point in health care institution management and optimization efforts^42^. By enhancing the operational efficiency and resource utilization of MRI systems, health care providers can significantly reduce operational costs, increase the quality of patient care, and improve treatment outcomes^43^. The MRI equipment of a hospital was chosen as the research object in this study, and a large medical institution was chosen as the case context to explore the operational benefits of its MRI equipment in depth.

The dataset for this research is derived from the Internet of Things (IoT)^44^, which enables data collectors to be affixed to medical imaging equipment^45^, specifically MRI systems. These collectors are proficient in capturing real-time operational metrics and standardizing otherwise nonstandardized data. To enrich the dataset, these data were integrated with existing health care information systems such as hospital information systems (HISs) and picture archiving and communication systems (PACSs)^46^, which provided a reliable database for the study. Twenty-four months of data from 1 January 2022 were selected as the study sample, covering the daily operation of each MRI to ensure the continuity, completeness and accuracy of the data.

### Evaluation results and analyses

#### Correlation analysis

**Figure 3:**
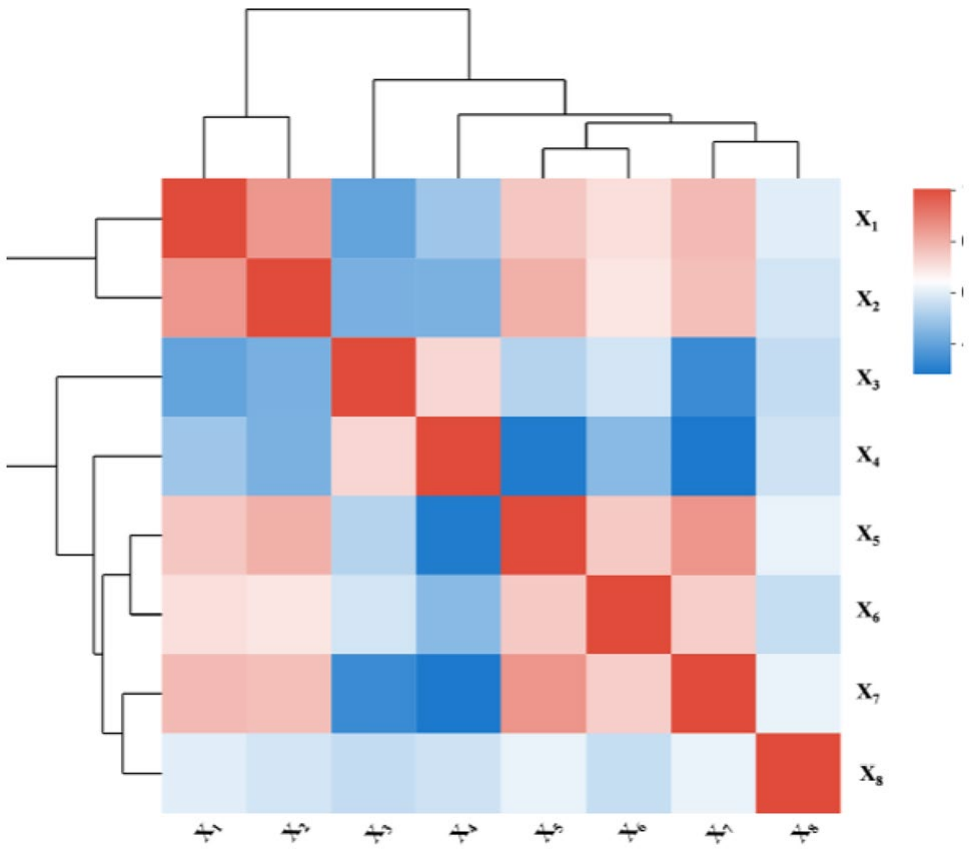
Indicator correlation.

Pearson correlation analysis^47^ is widely used to explore the degree of linear correlation between variables and to assist in selecting and optimizing model variables. The bivariate correlation analysis tool in SPSS software was used to analyse the correlation test and two-sided significance test p values of the input variables (Fig. 3), which showed that the range of correlation coefficients was concentrated at approximately 0.3–0.5. This indicates that the selected variables are moderately correlated and capable of explaining the target problem from different dimensions, and synergistic explanations can be generated when subsequent combinations are modelled. The ability to explain the target variables from different dimensions and generate complementary synergies in combined modelling lays the foundation for constructing the subsequent cone-constrained DEA.

#### Calculation of constraint weights

A judgement matrix was constructed using the expert scoring technique of the hierarchical analysis method to assess the relative importance of each input and output indicator in evaluating medical equipment effectiveness. First, several experts in the field of hospital equipment management were invited to rank the importance of the input and output indicators on a scale ranging from 1 to 9. The subsection was calculated according to the formula (Table 1), the CRs were 0.0287 and 0.0129, respectively, and the calculated CRs were less than 0.10, which verified the reliability of the results.

**Table 1:** AHP judgement matrix.

Input indicators				Output indicators			
1	2.1	1.5	1.8	1	1.3	2.5	1.8
0.476	1	0.333	0.5	0.769	1	3	2
0.666	3	1	2	0.4	0.333	1	0.5
0.555	2	0.5	1	0.555	0.5	2	1

In this study, the improved CRITIC method was used to determine the weights of indicators for evaluating medical equipment effectiveness (Table 2). First, the information entropy value of each indicator is calculated to obtain the matrix of the comparability coefficient between indicators. On this basis, the information correlation between each indicator and other indicators, i.e., the contradiction coefficient, is calculated, and the size of the independent information contained in each indicator is obtained according to the information quantity formula. The greater the amount of information, the greater the objective weight of the indicator; thus, the degree of influence of each evaluation indicator on the evaluation object and the calculation results are shown in the table.

**Table 2:** Results of the improvement of the critic.

Indicators	${X}_{1}$	${X}_{2}$	${X}_{3}$	${X}_{4}$	${X}_{5}$	${X}_{6}$	${X}_{7}$	${X}_{8}$
Information entropy	0.961	0.954	0.917	0.937	0.984	0.953	0.922	0.992
Comparative	0.206	0.205	0.363	0.190	0.207	0.186	0.292	0.135
Paradoxical	1.521	1.557	1.853	1.933	1.583	2.103	1.985	2.534
Volume of information	1.777	1.807	2.373	2.180	1.887	2.397	2.413	2.857
Weights	0.218	0.222	0.291	0.268	0.197	0.250	0.252	0.299

According to the results of the AHP evaluation, work saturation and equipment operating costs are most important among the input indicators, while among the output indicators, inspection revenue has the greatest weight. Therefore, decision-makers believe that the operation and cost of equipment directly affect the benefits of equipment use, and the most intuitive reflection of the benefits of the output is the equipment inspection revenue. The results of the CRITIC method show that the weights of the equipment operating cost, average cost per inspection, and waiting time for examination reports are relatively high, which indicates that these indicators have a greater degree of variability and a greater degree of independence from each other and, thus, have greater weights in the overall evaluation.

**Figure 4:**
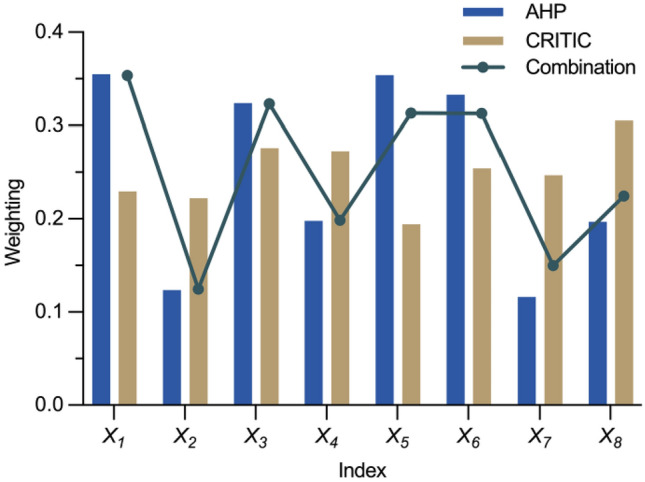
Combined weighting results.

There may be differences in the results obtained by different weight analysis methods, and game theory can fully consider the correlation between different weight vectors compared with simple combination algorithms such as direct averaging. After obtaining the weights of both the AHP and improved CRITIC methods, a game-theoretic combination of the weights is used. It views the two methods as two game parties with their respective weight vectors for different strategies. An objective function is established to minimize the deviation of the combined weight vector from the single-method weights, constraints are constructed, and the optimal objective is subsequently solved to obtain the combined weights (Fig. 4). The results show that the equipment operating cost has a high weight in both AHP and CRITIC, indicating that the indicator is not only important from the view of decision-makers but also provides unique information in data analysis, and the hybrid weights after the game-theoretic combination make the results more robust and effectively integrate the advantages of subjective and objective weight determination methods.

By calculating the combination weights of the input and output indicators, which can reflect the relative importance of each indicator, the constraint cone DEA model is constructed based on obtaining the mixed weights. Considering the research context and objectives of this article, a constant returns to scale (CRS) model was chosen to evaluate and compare the technical efficiency of different medical devices. First, according to the input and output indicators, the vertical and horizontal constraints are determined, constituting a reasonable efficiency evaluation interval. Lingo software is used to solve the constraint cone DEA model using linear programming techniques, which mainly include the objective function, constraint variable definitions, etc. By combining the actual input and output data of the equipment, the constraint cone calculations can map the efficiency of each piece of equipment into a multidimensional space and derive the relative efficiency scores, which reflect the relative efficiency level of each piece of equipment.

**Figure 5:**
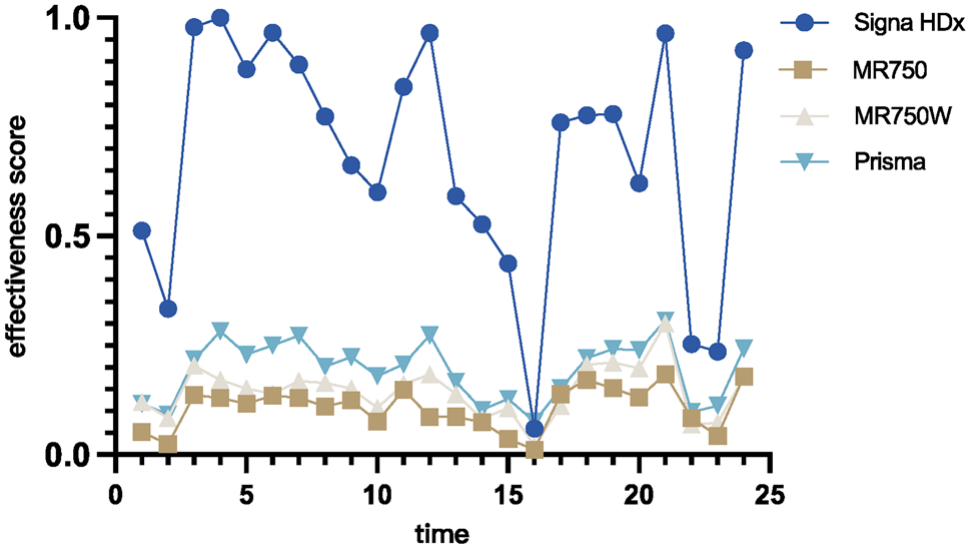
Effectiveness score.

After applying the constrained cone model calculations, the results of the change in the benefit scores for each MRI instrument for each month of the study period were obtained (Fig. 5). These benefit scores show the relative benefit level of the equipment at different points in time and take values ranging from 0 to 1, where 1 indicates that the equipment has reached the optimal level for all selected input‒output indicators.

As shown in the efficiency score obtained from the figure, equipment A presents a higher efficiency value, and there is a large difference between its score and those of the other three pieces of equipment, which is analysed for the input and output indicator data. In the distribution of the raw data of the index, the average daily working hours are relatively fixed, and the equipment utilization rate has relatively large fluctuations, indicating that the equipment efficiency could be improved. Due to the influence of the amount of equipment input, the purchase times, models, and functions of the different pieces of equipment are different, and the input costs of the four pieces of equipment have large differences, which has a large impact on the calculation results. As the price charged to patients for medical examination is relatively fixed, whether the equipment utilization rate is saturated intuitively affects the inspection revenue and cost‒benefit ratio of the equipment. Further exploring the correlation between the indicators and the benefit value, the following calculations were performed. The correlations between the equipment utilization rate (−0.58), equipment operating costs (−0.22) and average patient appointment waiting time (−0.57) and between the cost‒benefit ratio (−0.34) and the benefit values were found to be significantly negative, and the correlation between the inspection revenue and cost‒benefit ratio were significantly negative. A significant negative correlation and a positive correlation between inspection revenue (0.47) and benefit values indicate that increased revenue is associated with increased benefit values. Comprehensive analysis of equipment A revealed that, due to its earlier acquisition, it has lower equipment operating costs, it has the highest inspection revenue output, its benefit value is affected by large data fluctuations, and there is a significant gap between it and other pieces of equipment. According to our comprehensive understanding, equipment A has a high sweep speed, so it has a greater workload and generates greater benefits. Compared with the actual situation, the model calculation results and the actual situation are generally consistent, reflecting the applicability of the model.

**Figure 6:**
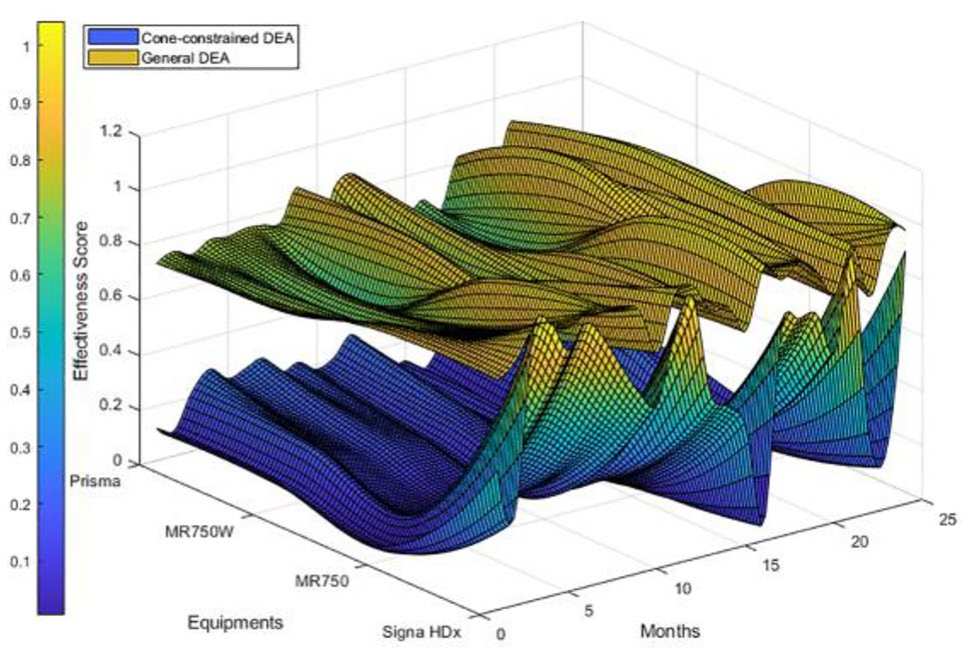
Comparison results.

To test the effectiveness of the constrained cone DEA model in evaluating medical equipment effectiveness, this study evaluated the monthly effectiveness scores of four MRI machines from January 2021 to December 2022 by using the traditional DEA model and the constructed cone-constrained DEA model and plotted the comparative results (Fig. 6).

Through comparative analysis, general DEA models usually only consider the linear constraint relationship between input and output indicators when evaluating equipment effectiveness and fail to fully consider the complex intercistronic relationships between multiple indicators. Cone-constrained DEA can consider the internal constraints between multiple effectiveness indicators, provide more robust and reasonable effectiveness intervals than the standard model, and help identify abnormal data points. Compared with traditional DEA, the constraint cone model can more accurately reflect the dynamic changes in equipment effectiveness, the score of which is more distinguishable, and can better identify inefficient equipment and optimization potential. The introduction of constraints can improve the robustness of DEA evaluations and provide a basis for subsequent equipment investment and management decisions.

#### LSTM model predictions

To further improve the accuracy and reliability of MRI equipment benefit prediction, this study proposes a new integrated evaluation method for medical equipment benefit prediction based on the attention mechanism Bi-LSTM model and the mixed weight constrained cone DEA model. This integrated approach fully exploits the sequence prediction advantages of the LSTM model. It combines the ability of the constrained cone DEA model with multicriteria decision-making methods, which helps to capture the dynamic changes in equipment benefits through learning and prediction of time-series data to more comprehensively enhance the reliable capture of the change patterns of medical equipment benefits and the prediction of reasonable intervals.

**Figure 7:**
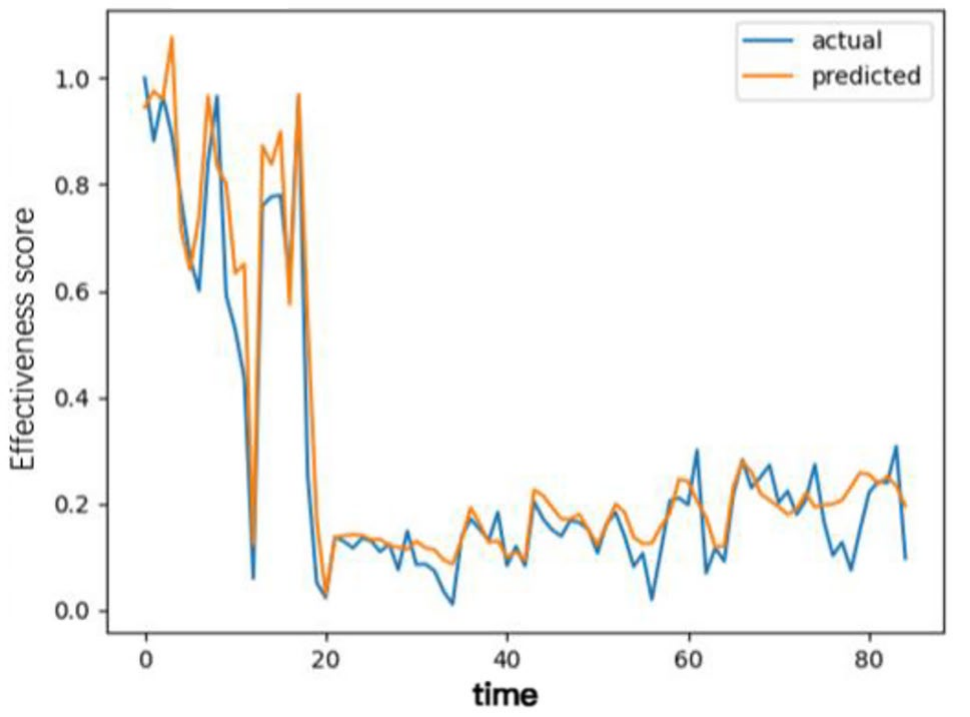
Prediction comparison results.

When constructing the model, this study preprocessed the equipment's historical benefit data, including normalization and missing value filling, to ensure the quality of the model input data and prevent numerical instability during training. At the same time, the time series data were divided into training and test sets to ensure the model's generalizability, and the Adam optimization algorithm was used to prevent model overfitting problems. The preprocessed training set data were input into the LSTM model for training. During training, the model continuously adjusts the weights and biases to minimize the error between the predicted and actual values through multiple iterations until the model converges or reaches a predetermined number of training rounds. In the actual prediction of equipment benefits, the mean square error (MSE) is used as the loss function; the mean square error (MSE) of the attention Bi-LSTM model is 0.0087, the coefficient of determination (R^2^) is 0.8759, and the mean absolute error (MAE) is 0.06274. Moreover, comparison results (Fig. 7) between the prediction and actual results are also obtained, which can intuitively show the model's performance in predicting MRI equipment benefits.

**Figure 8:**
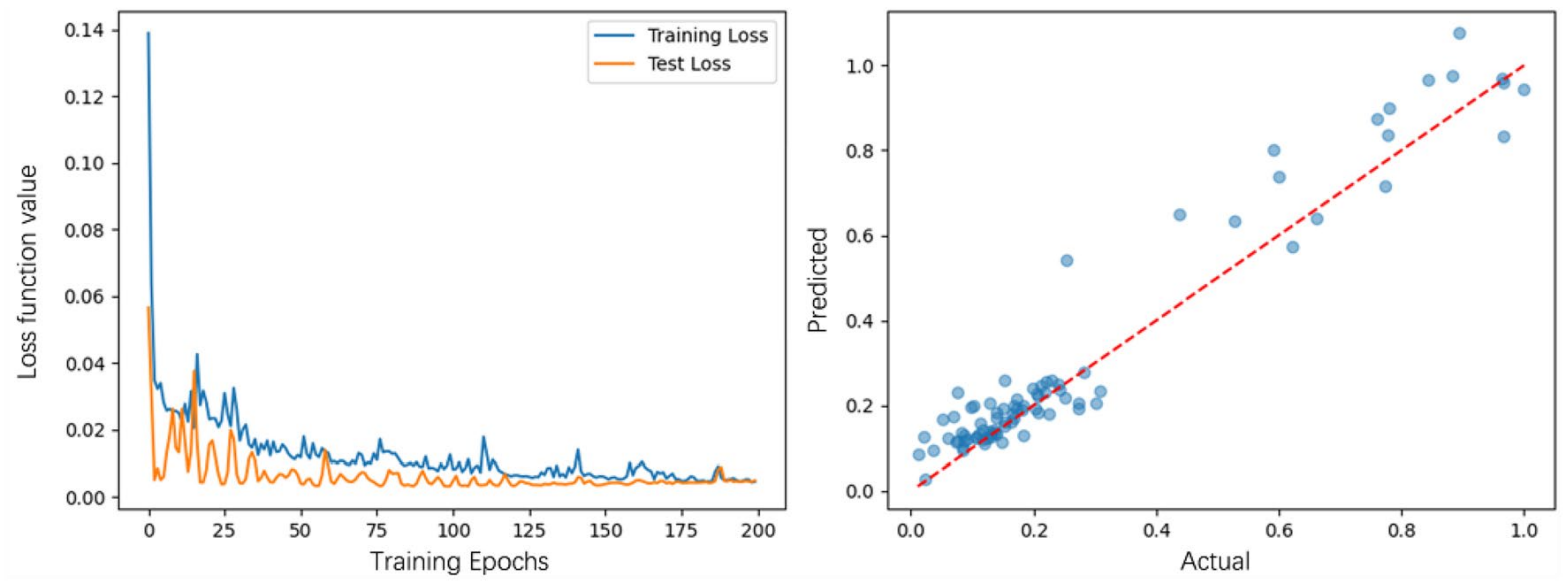
Loss function and scatter regression plot.

The loss function and the scatter regression plot of the prediction effect allow the change in the model's loss function during the training process to be visualized in two ways. After 200 rounds of training, the loss function of the mean square error of the model is reduced to 0.0077, which verifies the fitting effect of the model (Fig. 8). On the other hand, the scatter regression plot shows a positive correlation trend between the predicted and absolute benefit values.

In summary, in this study, through data preprocessing, feature engineering, and sequence construction of MRI equipment efficiency data for 24 consecutive months, the trained LSTM model performed well in predicting MRI equipment efficiency, with high predictive ability and interpretability. Therefore, the method can integrate time series characteristics and multi-indicator assessment capabilities to provide scientific and accurate trend predictions of equipment efficiency. This provides adequate decision support for further optimizing the utilization of MRI equipment resources and improving overall hospital efficiency.

## Discussion

The model in this article addresses the problem of evaluating the benefits of magnetic resonance imaging (MRI) equipment by constructing a comprehensive evaluation model, which is committed to overcoming the limitations of the traditional data envelopment analysis (DEA) model and incorporating the advantages of multiple evaluation methods. Traditional DEA models may lack the complete objectivity of indicator weights and reflect the objective differences in importance between indicators and the preferences of decision-makers. In contrast, the model in this article achieves exhaustive construction of the indicator hierarchy by introducing combination weights as constraints in the conical DEA framework, integrating the hierarchical analysis method (AHP) with the improved CRITIC method, balancing the opinions of experts with objective data analysis through game combination, enriching the decision-making process and enhancing the applicability and accuracy of the model to truly reflect the importance of each indicator. When dealing with temporal dynamics, a limitation of traditional DEA is its static nature, which is captured through the integration of the long short-term memory (LSTM) model, which introduces a predictive dimension to make the evaluation results more reflective of time-sensitive changes in benefits. However, there are limitations to this study; the temporal breadth of the current dataset and sample size limitations may restrict the generalizability of the model, and the nonconvexity of the variables could be investigated in more depth in the future when introducing ratio variables. Future research directions could include expanding the dataset to cover a wider range of medical device types and time horizons, and methodologically more sophisticated machine learning algorithms could be introduced to refine the model's predictive ability to further enhance its utility in strategic planning and operational optimization of medical device management.

## Conclusion

In summary, this study successfully constructed a new, effective, and comprehensive model for evaluating the effectiveness of medical equipment. In addition, introducing the LSTM model enhances the evaluation model's predictive ability and real-time performance to more accurately grasp and predict the development trend of equipment benefits, providing an adequate decision-making basis for the management and optimization of equipment. This innovative approach has essential theoretical and practical value for promoting medical equipment efficiency, improvement, and optimization. It is expected to be applied in a broader range of fields in the future, providing more scientific and accurate decision support for medical equipment management and optimization.

### Supplementary Information

Supplementary Information.

## Data Availability

Parts of the data generated or analyzed during this study are included in this published article [and its Supplementary Information File]. The datasets generated during and/or analysed during the current study are available from the corresponding author on reasonable request.
